# Supplementary material for: DdiA, an XRE family transcriptional regulator, is a co-regulator of the DNA damage response in Myxococcus xanthus
Source: J Bacteriol. 2025 Jul 3;207(7):e00184-25. doi: 10.1128/jb.00184-25 (PMC12288472; doi:10.1128/jb.00184-25)
Supplement: Supplemental material — Fig. S1 to S4, Tables S2 to S4, and supplemental references. [file jb.00184-25-s0001.pdf]

Supplementary Information

DdiA, an XRE family transcriptional regulator, is a co-regulator of the DNA damage response in *Myxococcus xanthus*

Jana Jung[1], Timo Glatter[2], Marco Herfurth[1] & Lotte Søgaard-Andersen[1, 3]

[1] Department of Ecophysiology, Max Planck Institute for Terrestrial Microbiology,

35043 Marburg, Germany

[2] Core Facility for Mass Spectrometry and Proteomics, Max Planck Institute for Terrestrial Microbiology, 35043 Marburg, Germany

This file contains:

- Supplementary Figures 1-4
- Supplementary Tables 2-4
- Supplementary References

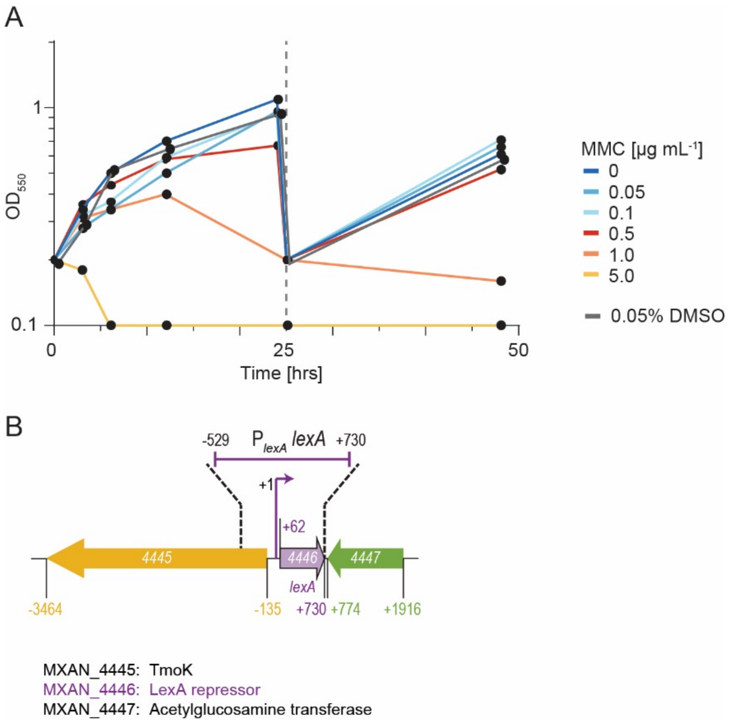

Figure S1. Determination of the highest non-lethal MMC concentration in *M. xanthus* and structure of *lexA* locus.

(A) Growth curves of WT *M. xanthus* in the presence of the indicated concentrations of MMC. Cells were grown in 1% CTT broth. Because MMC is dissolved in DMSO and the final DMSO concentration after addition to the growth medium is 0.05%, we also analyzed the growth of WT in the presence of 0.05% DMSO. At 24 hrs, the cultures were diluted into 1% CTT broth without MMC and DMSO. Data are from one representative experiment.

(B) Schematic of *lexA* locus. MXAN locus tags are included; genes are drawn to scale. +1 indicates the transcriptional start site of *lexA* (1). Numbers indicate the first and last nucleotide in start and stop codons, respectively relative to +1. Construct for the ectopic expression of *lexA* from its native promoter (P_{lexA}) is shown above. TmoK is a hybrid histidine protein kinase with a C-terminal GGDEF domain which lacks residues important for catalytic activity and c-di-GMP binding and is important for type IV pili-dependent motility (2).

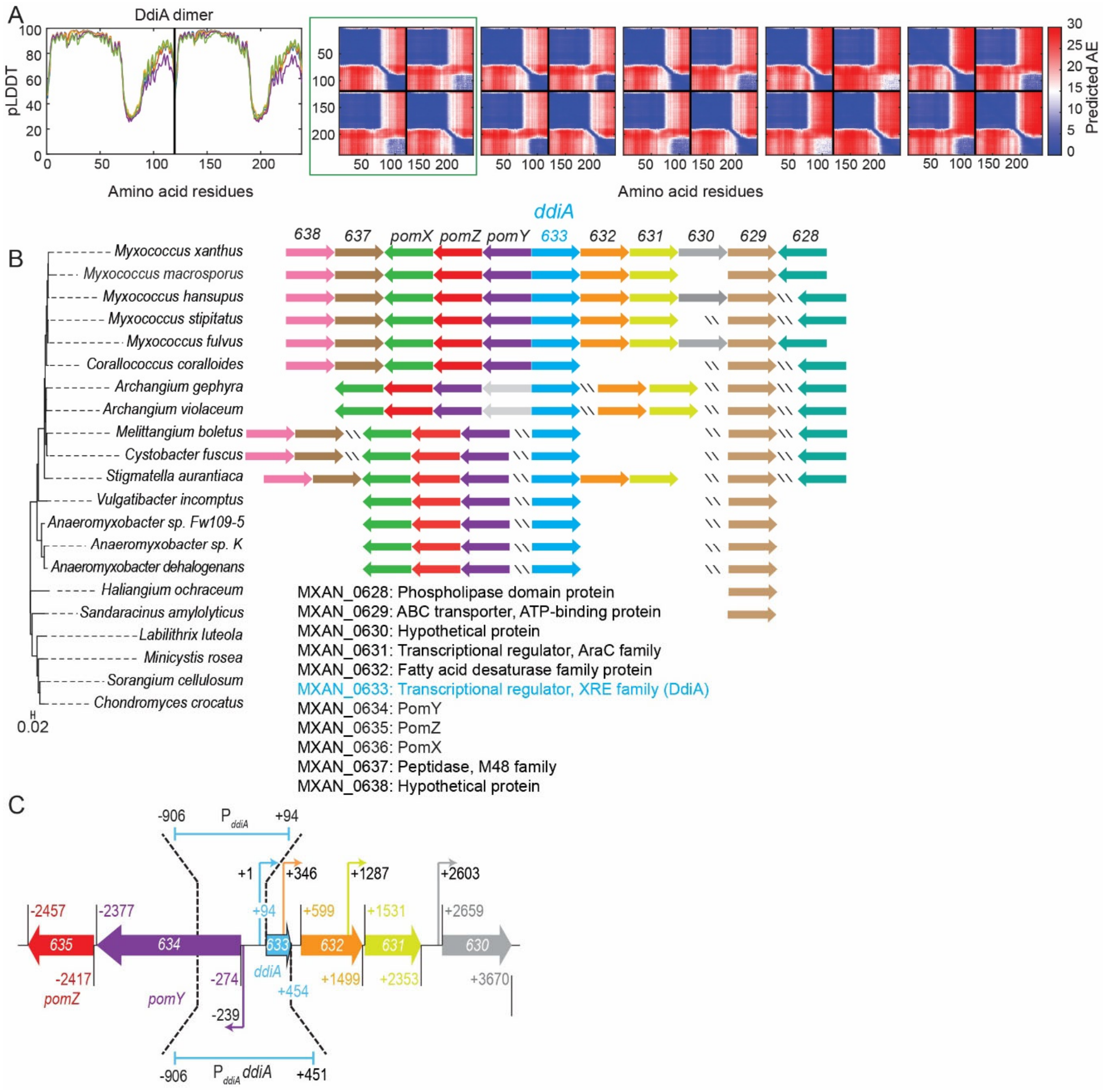

<u>Figure S2. Analysis of DdiA and the *ddiA* locus.</u>

(A) AlphaFold-Multimer structural model of DdiA dimer. Right panel, pLDDT plot for the five generated models of the DdiA dimer. Left panel, pAE plots for the five generated models of the DdiA dimer. The model highlighted by a green box was selected for further analysis.

(B) The *ddiA* locus is conserved in closely related myxobacteria. Transcription direction is indicated by the orientation of arrows with MXAN numbers indicated for the *ddiA* locus in *M. xanthus*.

(C) Detailed schematic of the *ddiA* locus. MXAN locus tags are included, genes are drawn to scale. +1 indicates the transcriptional start site of *ddiA* (1). Kinked arrows indicate transcriptional start sites relative to +1. Numbers indicate the first and last nucleotide in start and stop codons, respectively relative to +1. The blue bar labeled P_{ddiA} indicates the fragment upstream of the *ddiA* start codon used to generate the P_{ddiA}-*mCh* promoter fusion. The blue bar labeled P_{ddiA} *ddiA* indicates the fragment used for the ectopic expression of *ddiA-mCh* from the native P_{ddiA}.

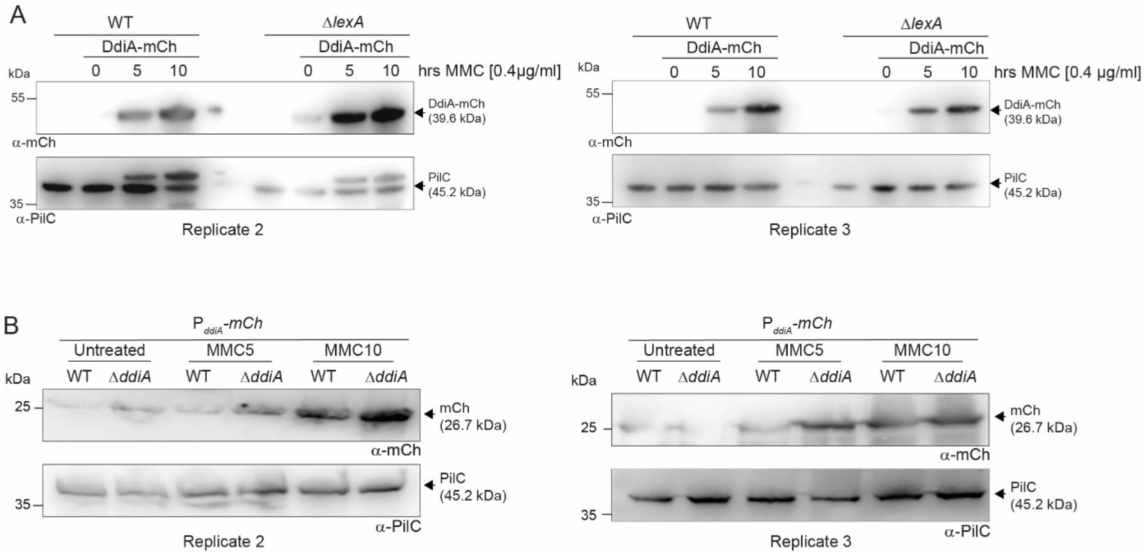

Figure S3. Analysis of DdiA-mCh abundance and P_{ddiA} expression.

(A) Immunoblot analysis of DdiA-mCh abundance. Cells were grown and DdiA-mCh abundance was quantified as in Fig. 3E. The two blots represent the two replicates, which together with the replicate in Fig. 3E, were used to quantify DdiA-mCh by immunoblotting.

(B) Immunoblot analysis of P_{ddiA} expression. P_{ddiA} was fused to a promoterless *mCh*. Cells were grown and mCh abundance was quantified as in Fig.3G. The two blots represent the two replicates, which together with the replicate in Fig.3G, were used to quantify mCh by immunoblotting.

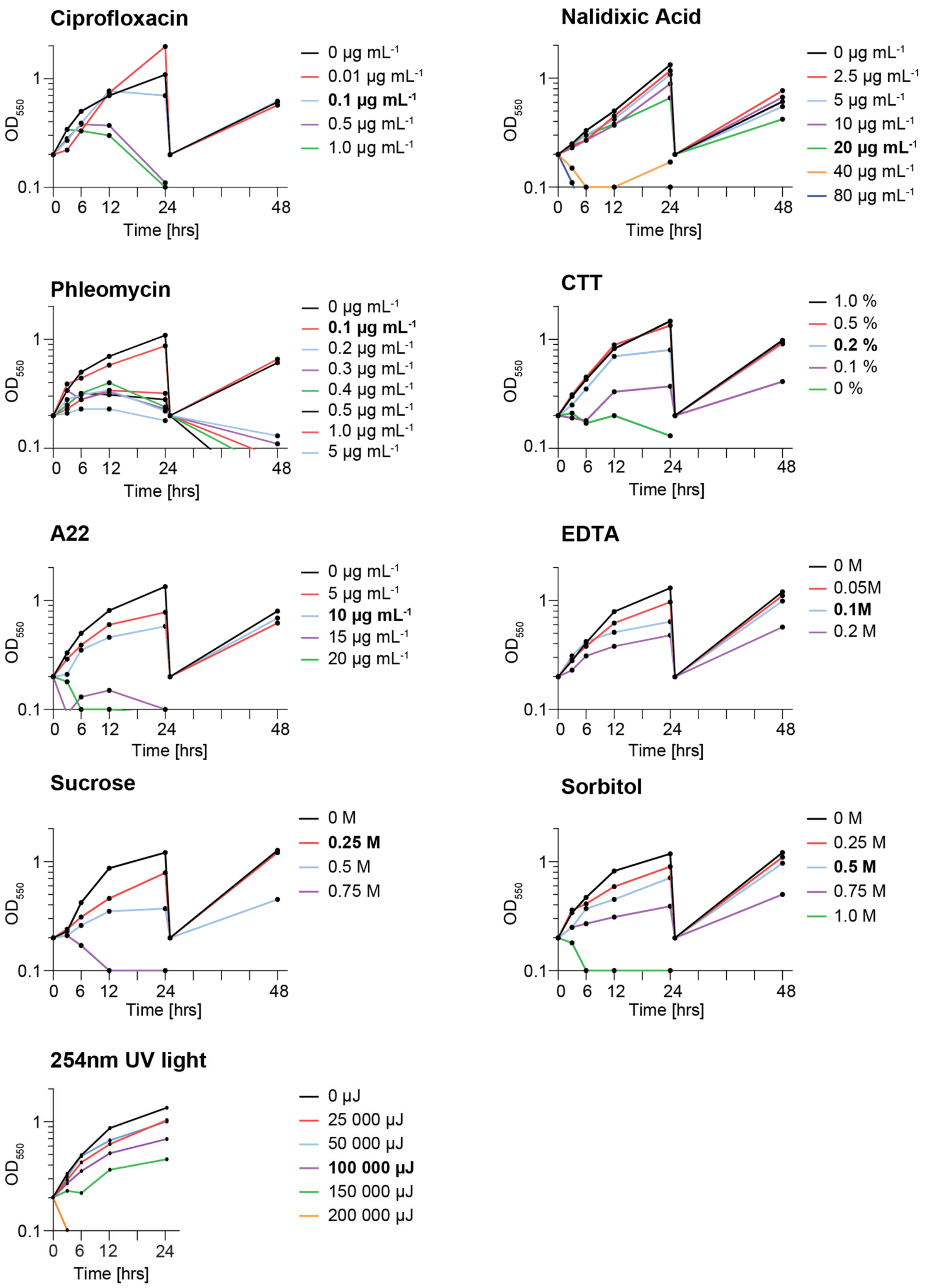

Figure S4. Growth curves of *M. xanthus* WT in the presence of various stressors.

Growth curves of WT *M. xanthus* in the presence of the indicated stressors. For all stressors except UV light, cells were grown in 1% CTT broth in the presence of the indicated stressor. At 24 hrs, the cultures were diluted into 1% CTT broth without the stressor. In the UV light experiment, cells were exposed to a single dose of 254nm UV light in 1% CTT (t=0hrs), and

then grown in suspension culture in the dark in the absence of UV light. Data are from one representative experiment. Bold marks the conditions used in the experiments in Fig. 5D.

Table S2. *M. xanthus* strains used in this study.

Strain	Genotype	Reference
DK1622	Wildtype	(3)
SA9439	Δ*lexA*	This study
SA9440	Δ*lexA*, *attB*::pJJ51 (P_{lexA} *lexA*)	This study
SA9405	Δ*ddiA*	This study
SA9406	Δ*ddiA*, *attB*::pJJ35 (P_{ddiA} *ddiA*)	This study
SA9409	Δ*ddiA*, *attB*::pJJ37 (P_{ddiA} *ddiA-mCh*)	This study
SA9433	*ddiA*::*ddiA-mCh*	This study
SA9438	*ddiA*::*ddiA-mCh* Δ*lexA*	This study
SA9441	*attB*::P_{ddiA}-*mCh* (pJJ50)	This study
SA9442	*attB*::P_{ddiA}-*mCh* (pJJ50) Δ*ddiA*	This study

Table S3. Plasmids used in this study.

Plasmid	Description	Reference
pSW105	*attP*, P_{pilA}, Km^R	(4)
pSWU30	*attP* Tet^R	(5)
pBJ114	*galK*; Km^R	(6)
pJJ34	pBJ114; for generation of in-frame deletion of *ddiA* (*MXAN_0633*)	This study
pJJ35	For integration at *attB* and expression of *ddiA* from P_{ddiA}	This study
pJJ37	For integration at *attB* and expression of *ddiA-mCh* from P_{ddiA}	This study
pJJ38	For integration of *ddiA-mCh* at the native site	This study
pJJ47	pBJ114; For generation of in-frame deletion of *lexA* (*MXAN_4447*)	This study
pJJ50	For integration at *attB* and expression of *mCh* from from P_{ddiA}	This study
pJJ51	For integration at *attB* and expression of *lexA* from P_{lexA}	This study

Table S4. Oligonucleotides used in this study.

Name	Sequence (5´ to 3´)	Brief description
JJ13_DdiA_A_HindIII	GCGAAGCTTCTCCAGCTCCGCGACCTCACGGCGC	For construction of pJJ34
JJ14_DdiA_B	CGCCACCTGCCCTCCAATGAGGGTTGCCAGTC	
JJ15_DdiA_C	CCCTCATTGGAGGGCAGGTGGCGAACGCGCT	
JJ16_DdiA_D_XbaI	GCGTCTAGATGCCAGACCTGGCCCAGCGTCTCG	
JJ53_DdiA_E	GGATGCGCCCCACGTCCAGCAG	
JJ54_DdiA_F	CAGCGCGGGCTTCCACACGATG	
JJ55_DdiA_G	CGCGGTCCGCGCGGCGCG	
JJ56_DdiA_H	GGCGAAGACGCGGTGCGTCC	
JJ45_LexA_A_HindIII	GCGAAGCTTGCTCCTTGTCCTTGCGCAGCTGCG	For construction of pJJ47
JJ46_LexA_B	TGCGGCCGCCGCTCTTCCATGCGCTCCCTC	
JJ47_LexA_C	ATGGAAGAGCGGCGGCCGCACCCCGTAGTC	
JJ48_LexA_D_XbaI	GCGTCTAGACCACACCGCGGACGTGCTCG	
JJ49_LexA_E	GGACGAAGAGCGCCTCCACG	
JJ50_LexA_F	CCTCGAAGCAGCGCTACCCGG	
JJ51_LexA_G	GCGCGAGATTCTCAGCTTCATCG	
JJ52_LexA_H	CATCGACTGCCCCTTCACGC	
JJ17_EcoRI_up_633_fwd	GCGGAATTCCTGCAGGAAGCGCTGCGCCTCACGCGCC	For construction of pJJ37
JJ20_633_Linker_rev	GGCGGCGGATCTGGCGGACGTCTCGAGCGCGTTCGCCACCTGC	
JJ21_Linker_mCh_fwd	TCCGCCAGATCCGCCGCCGGCTCCGGCATGGTGAGCAAGGGCG	
JJ22_HindIII_mCh_rev	GCGAAGCTTTTACTTGTACAGCTCGTCCATGCC	
JJ17 EcoRI_up_633_fwd	GCGGAATTCCTGCAGGAAGCGCTGCGCCTCACGCGCC	For construction of pJJ35
JJ18 HindIII_633_rev	GCGAAGCTTCTACGTCTCGAGCGCGTTCGCCACCTGC	
JJ59_LexA_A_EcoRI	GCGGAATTCGCTCCTTGTCCTTGCGCAGCTGCG	For construction of pJJ51
JJ60_LexA_rev_HindIII	CGCAAGCTTCTACGGGGTGCGGCCGCCCTGGAGC	
JJ23_HindIII_up_633_fwd	GCGAAGCTTCCGCGCAGGCCGAATTTTCGGCTTCCGCGC	For construction of pJJ38
JJ24_down_0633_mCh_rev	CCGCTGTATCTTACTTGTACAGCTCGTCC	
JJ25_mCh_down_0633_fwd	GTACAAGTAAGATACAGCGGAGACCCGGCC	
JJ26_DdiA_D_XbaI	GCGTCTAGATGCCAGACCTGGCCCAGCGTCTCG	
JJ17_EcoRI_up_633_fwd	GCGGAATTCCTGCAGGAAGCGCTGCGCCTCACGCGCC	For construction of ppJJ50
JJ57_PddiA_mCh_fwd	CCGCACGCCATGGTGAGCAAGGGCGAGGA	
JJ58_mCh_PddiA_rev	GCTCACCATGGCGTGCGGAAAACCTGTCG	
JJ59_HindIII_stop_mCh	GCGAAGCTTTTACTTGTACAGCTCGTCCATGCC	
JJ77_TrpA_fwd_3	GATTGGCGTGGCGTTCAG	RT-qPCR for *trpA*
JJ78_TrpA_rev_3	GGGGACATCGCTTGCGTA	
JJ81_UvrA_fwd_2	ACAGATGGAGAAGCCGAAG	RT-qPCR for *uvrA*
JJ82_UvrA_rev_2	ATAAAGCACCCGCAGGTAG	
JJ87_DdiA_fwd_2	GGCAACCCTCATTGGAAACG	RT-qPCR for *ddiA*
JJ88_DdiA_rev_2	CGTCCGACGAGCAGTTGA	
JJ91_DnaE2_fwd_1	CGCCTCATTGGATGACTTG	RT-qPCR for *dnaE2*
JJ92_DnaE2_rev_1	GCTCAATCTGCTTCACCAC	
JJ97_RecX_fwd_1	AGGACCCGACAAGAAGAAGGAG	RT-qPCR for *recX*
JJ98_RecX_rev_1	AACTTGATGGAGCGGTCCAC	

Supplementary References

1. Kuzmich S, Blumenkamp P, Meier D, Szadkowski D, Goesmann A, Becker A, Søgaard-Andersen L. 2022. CRP-like transcriptional regulator MrpC curbs c-di-GMP and 3',3'-cGAMP nucleotide levels during development in *Myxococcus xanthus*. mBio 13:e00044-22.

2. Skotnicka D, Petters T, Heering J, Hoppert M, Kaever V, Søgaard-Andersen L. 2015. c-di-GMP regulates type IV pili-dependent-motility in *Myxococcus xanthus*. J Bacteriol 198:77-90.

3. Kaiser D. 1979. Social gliding is correlated with the presence of pili in *Myxococcus xanthus*. Proc Natl Acad Sci U S A 76:5952-6.

4. Jakovljevic V, Leonardy S, Hoppert M, Søgaard-Andersen L. 2008. PilB and PilT are ATPases acting antagonistically in type IV pili function in *Myxococcus xanthus*. J Bacteriol 190:2411-2421.

5. Wu S, Kaiser D. 1997. Regulation of expression of the *pilA* gene in *Myxococcus xanthus*. J Bacteriol 179:7748-7758.

6. Julien B, Kaiser AD, Garza A. 2000. Spatial control of cell differentiation in *Myxococcus xanthus*. Proc Natl Acad Sci U S A 97:9098-9103.
